# Sequential boundaries approach in clinical trials with unequal allocation ratios

**DOI:** 10.1186/1471-2288-6-1

**Published:** 2006-01-13

**Authors:** Peyman Jafari, Seyyed Mohammad Taghi Ayatollahi, Javad Behboodian

**Affiliations:** 1 Department of Biostatistics and Epidemiology, Faculty of Health, Shiraz University of Medical Sciences, Shiraz, Iran; 2 Department of Statistics, Faculty of Science, Shiraz University, Shiraz, Iran

## Abstract

**Background:**

In clinical trials, both unequal randomization design and sequential analyses have ethical and economic advantages. In the single-stage-design (SSD), however, if the sample size is not adjusted based on unequal randomization, the power of the trial will decrease, whereas with sequential analysis the power will always remain constant. Our aim was to compare sequential boundaries approach with the SSD when the allocation ratio (R) was not equal.

**Methods:**

We evaluated the influence of R, the ratio of the patients in experimental group to the standard group, on the statistical properties of two-sided tests, including the two-sided single triangular test (TT), double triangular test (DTT) and SSD by multiple simulations. The average sample size numbers (ASNs) and power (1-β) were evaluated for all tests.

**Results:**

Our simulation study showed that choosing R = 2 instead of R = 1 increases the sample size of SSD by 12% and the ASN of the TT and DTT by the same proportion. Moreover, when R = 2, compared to the adjusted SSD, using the TT or DTT allows to retrieve the well known reductions of ASN observed when R = 1, compared to SSD. In addition, when R = 2, compared to SSD, using the TT and DTT allows to obtain smaller reductions of ASN than when R = 1, but maintains the power of the test to its planned value.

**Conclusion:**

This study indicates that when the allocation ratio is not equal among the treatment groups, sequential analysis could indeed serve as a compromise between ethicists, economists and statisticians.

## Background

One of the key reasons for using sequential methods, instead of single-stage design (SSD), in planning clinical trials is that the expected number of patients is decreased while maintaining the pre-specified significance level and power. Sequential designs have become common practice in interim monitoring of clinical trials because of their ethical and economic advantages. Nowadays, investigators planning a clinical trial have a wide range of sequential methods available to choose. These methods can be categorized in two different types: boundaries approach and repeated significance tests [1-6]. In this paper we have only considered boundaries approach, including triangular test (TT) and double triangular test (DTT), because of their interesting properties [2,7-9]. Sebille and Bellissant presented a nice account of the properties of TT and DTT [7,8,10]. However, the properties of these methods, in some cases, are still unknown. In particular, situations not dealt with in previous articles are sequential designs with unequal randomization ratios.

In a randomized controlled clinical trial with two treatments, it is a standard practice to have approximately equal-sized treatment groups since it maximizes the statistical power for a given total sample size. However, there are several research papers with topics on unequal randomization that have demonstrated the efficiency of this method in clinical trials [11-16]. They showed that in some trials, which compare a new treatment against a standard, using unequal randomization could be helpful from the ethical and economic viewpoints. Yet there is no consensus between ethicists and economists on the issue. On the other hand, since sequential designs have ethical and economic advantages per se, it seems reasonable to use these methods when investigators decide to randomize patients to the experimental and standard treatment groups in unequal ratios.

Hence, the purpose of this study is to assess the effect of unequal randomization on the statistical properties of TT, DTT and SSD adjusted for unequal allocation ratio (SSD_adj_) by multiple simulation, and SSD using the formulas used by Pocock [16]. In all of these methods the power and average sample size numbers (ASNs) were computed when the patients were allocated to the experimental and standard treatments in different ratios.

## Methods

We follow notations similar to that used by Sebille and Bellissant [8]. Let θ be a measure of the difference between the experimental and standard treatments. The clinical trial can be viewed as a test of the null hypothesis of no treatment difference H_0 _(θ = 0) against the alternative that there is a difference H_1 _(θ ≠ 0). This parameter is designed such that θ = 0 when treatments are equivalent, θ > 0 (H1+
 MathType@MTEF@5@5@+=feaafiart1ev1aaatCvAUfKttLearuWrP9MDH5MBPbIqV92AaeXatLxBI9gBaebbnrfifHhDYfgasaacH8akY=wiFfYdH8Gipec8Eeeu0xXdbba9frFj0=OqFfea0dXdd9vqai=hGuQ8kuc9pgc9s8qqaq=dirpe0xb9q8qiLsFr0=vr0=vr0dc8meaabaqaciaacaGaaeqabaqabeGadaaakeaacqqGibasdaqhaaWcbaGaeeymaedabaGaee4kaScaaaaa@2FB6@) when the experimental treatment is better than the Standard one, and θ < 0 (H1-
 MathType@MTEF@5@5@+=feaafiart1ev1aaatCvAUfKttLearuWrP9MDH5MBPbIqV92AaeXatLxBI9gBaebbnrfifHhDYfgasaacH8akY=wiFfYdH8Gipec8Eeeu0xXdbba9frFj0=OqFfea0dXdd9vqai=hGuQ8kuc9pgc9s8qqaq=dirpe0xb9q8qiLsFr0=vr0=vr0dc8meaabaqaciaacaGaaeqabaqabeGadaaakeaacqqGibasdaqhaaWcbaGaeeymaedabaGaeeyla0caaaaa@2FBA@) when the experimental treatment is worse.

The trial considered here only involves the comparison of two normally distributed responses in two-sided tests. We defined the effect size as the difference between treatments in units of standard deviation, θ_R _= (μ_2_-μ_1_)/σ where μ_1 _and μ_2 _are the means for the standard and experimental groups, respectively, and σ is the common standard deviation (σ_1 _= σ_2 _= σ).

### Single stage design (SSD)

The traditional statistical approach in the analysis of clinical trials is SSD with equal patients in each group. In this method, the sample size is computed at the design phase based on the significance level (α), difference of clinical interest (θ_R_), and power (1-β). In a two-group comparative study where the response measure is normally distributed, the total sample size formula would be:

N = 4(Z1−α/2+Z1−βθR)2     (1),
 MathType@MTEF@5@5@+=feaafiart1ev1aaatCvAUfKttLearuWrP9MDH5MBPbIqV92AaeXatLxBI9gBaebbnrfifHhDYfgasaacH8akY=wiFfYdH8Gipec8Eeeu0xXdbba9frFj0=OqFfea0dXdd9vqai=hGuQ8kuc9pgc9s8qqaq=dirpe0xb9q8qiLsFr0=vr0=vr0dc8meaabaqaciaacaGaaeqabaqabeGadaaakeaacqqGobGtcqqGGaaicqqG9aqpcqqGGaaicqqG0aandaqadaqaamaalaaabaGaeeOwaO1aaSbaaSqaaiabbgdaXiabgkHiTiabeg7aHjabc+caViabikdaYaqabaGccqGHRaWkcqqGAbGwdaWgaaWcbaGaeeymaeJaeyOeI0IaeqOSdigabeaaaOqaaiabeI7aXnaaBaaaleaacqqGsbGuaeqaaaaaaOGaayjkaiaawMcaamaaCaaaleqabaGaeGOmaidaaOGaaCzcaiaaxMaadaqadaqaaiabigdaXaGaayjkaiaawMcaaiabcYcaSaaa@484B@

where θR=μ2−μ1σ
 MathType@MTEF@5@5@+=feaafiart1ev1aaatCvAUfKttLearuWrP9MDH5MBPbIqV92AaeXatLxBI9gBaebbnrfifHhDYfgasaacH8akY=wiFfYdH8Gipec8Eeeu0xXdbba9frFj0=OqFfea0dXdd9vqai=hGuQ8kuc9pgc9s8qqaq=dirpe0xb9q8qiLsFr0=vr0=vr0dc8meaabaqaciaacaGaaeqabaqabeGadaaakeaacqaH4oqCdaWgaaWcbaGaeeOuaifabeaakiabg2da9maalaaabaGaeqiVd02aaSbaaSqaaiabikdaYaqabaGccqGHsislcqaH8oqBdaWgaaWcbaGaeGymaedabeaaaOqaaiabeo8aZbaaaaa@3943@ and Z_α _is the upper 100α% percentile of *N*(0,1), that is, α = 1 - Φ(Z_α_). *Z*_1-β _is defined similarly.

If N_E _and N_S _denote the numbers of patients assigned to experimental and standard treatments with N_E _+ N_S _= N being fixed and r = NEN
 MathType@MTEF@5@5@+=feaafiart1ev1aaatCvAUfKttLearuWrP9MDH5MBPbIqV92AaeXatLxBI9gBaebbnrfifHhDYfgasaacH8akY=wiFfYdH8Gipec8Eeeu0xXdbba9frFj0=OqFfea0dXdd9vqai=hGuQ8kuc9pgc9s8qqaq=dirpe0xb9q8qiLsFr0=vr0=vr0dc8meaabaqaciaacaGaaeqabaqabeGadaaakeaacqqGYbGCcqqGGaaicqqG9aqpcqqGGaaidaWcaaqaaiabb6eaonaaBaaaleaacqqGfbqraeqaaaGcbaGaeeOta4eaaaaa@3443@ denotes the proportion on the experimental treatment, then the power under H_1 _is given by [16]:

power = Φ{2[Φ−1(1−α)+Φ−1(1−β)]×r(1−r)−Φ−1(1−α)}     (2).
 MathType@MTEF@5@5@+=feaafiart1ev1aaatCvAUfKttLearuWrP9MDH5MBPbIqV92AaeXatLxBI9gBaebbnrfifHhDYfgasaacH8akY=wiFfYdH8Gipec8Eeeu0xXdbba9frFj0=OqFfea0dXdd9vqai=hGuQ8kuc9pgc9s8qqaq=dirpe0xb9q8qiLsFr0=vr0=vr0dc8meaabaqaciaacaGaaeqabaqabeGadaaakeaacqqGWbaCcqqGVbWBcqqG3bWDcqqGLbqzcqqGYbGCcqqGGaaicqqG9aqpcqqGGaaicqqHMoGrdaGadaqaaiabbkdaYmaadmaabaGaeuOPdy0aaWbaaSqabeaacqaIXaqmaaGccqGGOaakcqaIXaqmcqGHsislcqaHXoqycqGGPaqkcqGHRaWkcqqHMoGrdaahaaWcbeqaaiabgkHiTiabigdaXaaakiabcIcaOiabigdaXiabgkHiTiabek7aIjabcMcaPaGaay5waiaaw2faaiabgEna0oaakaaabaGaeeOCaiNaeeikaGIaeeymaeJaeyOeI0IaeeOCaiNaeeykaKcaleqaaOGaeyOeI0IaeuOPdy0aaWbaaSqabeaacqGHsislcqaIXaqmaaGccqGGOaakcqaIXaqmcqGHsislcqaHXoqycqGGPaqkaiaawUhacaGL9baacaWLjaGaaCzcamaabmaabaGaeGOmaidacaGLOaGaayzkaaGaeiOla4caaa@6513@

In this formula Φ(·) denotes the cumulative function of the standard normal distribution *N*(0,1).

However, if the investigator decides to allocate patients in unequal ratio and aims to achieve the pre-specified power, then the total sample size for SSD should be adjusted by a factor dependent on the allocation ratio. Therefore, the total sample size for SSD_adj _is equal to [2]:

Nadj=(R+1)2R(Z1−α/2+Z1−βθR)2=(R+1)2N4R     (3),
 MathType@MTEF@5@5@+=feaafiart1ev1aaatCvAUfKttLearuWrP9MDH5MBPbIqV92AaeXatLxBI9gBaebbnrfifHhDYfgasaacH8akY=wiFfYdH8Gipec8Eeeu0xXdbba9frFj0=OqFfea0dXdd9vqai=hGuQ8kuc9pgc9s8qqaq=dirpe0xb9q8qiLsFr0=vr0=vr0dc8meaabaqaciaacaGaaeqabaqabeGadaaakeaacqqGobGtdaWgaaWcbaGaeeyyaeMaeeizaqMaeeOAaOgabeaakiabg2da9maalaaabaGaeiikaGIaeeOuaiLaee4kaSIaeeymaeJaeeykaKYaaWbaaSqabeaacqqGYaGmaaaakeaacqqGsbGuaaWaaeWaaeaadaWcaaqaaiabbQfaAnaaBaaaleaacqqGXaqmcqGHsislcqaHXoqycqGGVaWlcqaIYaGmaeqaaOGaey4kaSIaeeOwaO1aaSbaaSqaaiabbgdaXiabgkHiTiabek7aIbqabaaakeaacqaH4oqCdaWgaaWcbaGaeeOuaifabeaaaaaakiaawIcacaGLPaaadaahaaWcbeqaaiabikdaYaaakiabg2da9maalaaabaGaeiikaGIaeeOuaiLaee4kaSIaeeymaeJaeeykaKYaaWbaaSqabeaacqqGYaGmaaGccqqGobGtaeaacqqG0aancqqGsbGuaaGaaCzcaiaaxMaadaqadaqaaiabiodaZaGaayjkaiaawMcaaiabcYcaSaaa@5B16@

where R is the ratio of patients in the experimental group to the standard group or the reverse ratio.

Once the data have been collected, the statistical analysis is conducted. Based on the SSD or SSD_adj _we cannot stop an ongoing trial before inclusion of a predetermined sample size, even if the early data show a clear difference between treatments.

### Boundaries approach: triangular and double triangular tests (TT and DTT)

**Figure 1:**
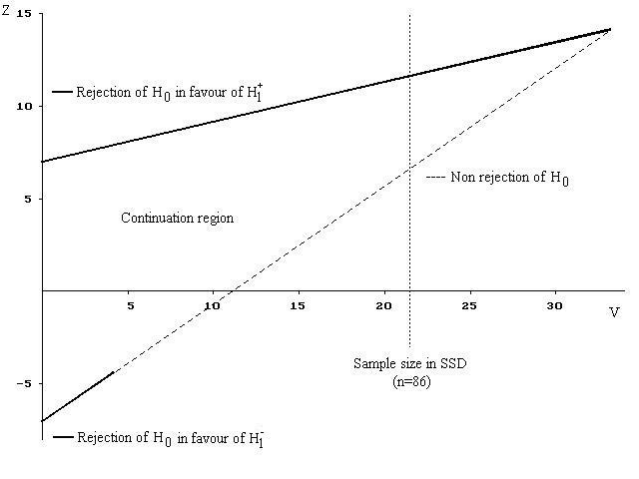
Stopping boundaries based on the TT for α = 0.05, β = 0.05 and R = 1 with θ_R _= 0.7.

**Figure 2:**
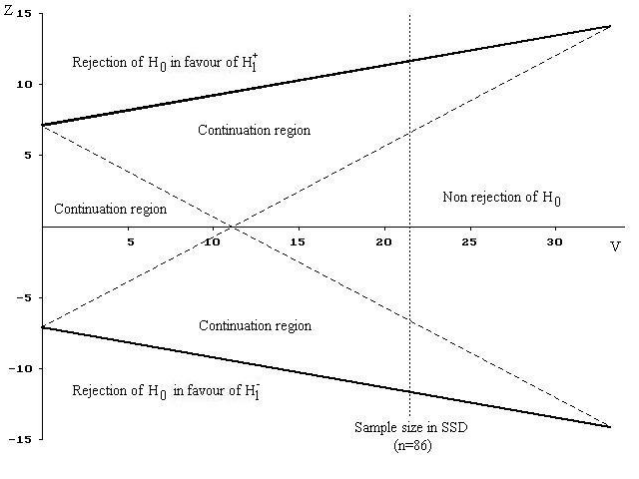
Stopping boundaries based on the DTT for α = 0.05, β = 0.05 and R = 1 with θ_R _= 0.7.

Sequential boundaries approach, the TT and DTT, permit repeated statistical analyses to be performed throughout the trial recruitment period in order to allow for early termination of a trial while maintaining a pre-specified α and β level. This reduction in sample size has ethical and economic advantages [8]. From the ethical viewpoint, this reduction minimizes the number of patients who will be given an inferior or ineffective treatment. Moreover, from the economic viewpoint, it leads to saving in time and resources. As shown in Figures 1 and 2, the TT and DTT are based on the two-perpendicular axes [2,9]. These two axes are two sample statistics that play particularly important role in the investigation of θ and are fundamental to sequential trials. The vertical axis is a cumulative measure of the advantage of the experimental treatment, and will be denoted by Z (efficient score for that calculated under the null hypothesis). The horizontal axis, denoted by V, indicates the amount of information about θ contained in Z (Fisher's information) and it will increase as the trial progresses [7]. The straight lines, the boundaries of the tests, delineate a continuation and stopping region. The equations of the straight line boundaries depend on the values of the benefit to detect, and α and β, as well as on the frequency of the analyses, defined in terms of the number of patients included between two analyses [2]. At each analysis, the two statistics V and Z are calculated from all the data collected since the beginning of the study and a point (V, Z) is defined on the sequential plan. The consecutive points define a sample path from the left to the right of the sequential plan. As long as the sample path stays within the two boundaries, the study is continued and new patients are included. When the sample path crosses one of the boundaries, the trial is stopped.

The triangular tests can be categorized in two classes based on their power function. The power function, denoted by C (θ), is defined as the probability that H_0 _is rejected when the parameter θ is true. When the true treatment difference is θ, C^+ ^(θ) and C^- ^(θ) are the probability of reaching the conclusion that the experimental treatment is significantly better and worse than the standard, respectively. Based on this definition two alternative power requirements will be specified: power requirement I and power requirement II. TT is designed to satisfy power requirement I. In this situation C^+^(θ_R_) = 1-β but no specification is made for C^- ^(-θ_R_) and also C^- ^(θ_R_) is usually negligible. On the other hand, DTT is designed to satisfy power requirement II. In this situation, C^+^(θ_R_) = C^- ^(-θ_R_) = 1-β and both C^+^(-θ_R_) and C^- ^(θ_R_) are negligible [2,17].

### Simulation study

We studied the ASN for the TT and DTT by multiple simulations in PEST3 [17]. Our simulation design was very similar to that used by Sebille and Bellissant [8]. For each studied situation, we generated 30,000 independent comparative trials in which patient responses were drawn from a normal distribution with mean μ_1 _(mean response in standard group) equal to 10 and the standard deviation equal to 5. The influences of different values of β and θ_R _(μ_2_) on the statistical properties of all tests were evaluated. The total number of patients at each interim analysis (n) was equal to 12. We also evaluated the influence of the allocation ratio (R) on the statistical properties. R is defined as the ratio of the patients in the experimental group to the standard group. Namely, we chose two different values for β(0.05 and 0.1), seven values for θ_R _(0.4, 0.5, 0.6, 0.7, 0.8, 0.9 and 1.0), one value for n (n = 12) and two values for R (1 and 2). The value of α was set to 0.05 for all simulated trials. We also calculated the required sample size for SSD and SSD_adj _for the same values of θ_R_, R, β and α as for TT and DTT by Formulas (1) and (3), respectively. Moreover, we simulated the required ASN and power for the TT (n = 12), DTT (n = 12), and two-sided SSD_adj _for different values of R, when θ_R _= 0.7 and β = α = 0.05. For SSD, the required sample size and power were calculated for the same value of R, θ_R_, α and β using Formulas (1) and (2).

## Results

Table 1 shows the ASN of patients required to reach a conclusion under H_0_, H_1 _and θ = θ_R_/2 for different values of θ_R _and β for the SSD when R = 1, SSD when R = 2 (SSD_adj_), TT when R = 1 and when R = 2, and DTT when R = 1 and when R = 2. The ASNs under H_0_, H_1 _and θ = θ_R_/2 were smaller for all sequential tests than for SSD (R = 1), whatever values of θ_R_, β and R were considered. Indeed, as compared with the SSD (R = 1), there were decreases of approximately 39% and 20% under H_0_, 29% and 28% under H_1_, and 14% and 12% under θ = θ_R_/2 in the ASNs for the TT (R = 2) and DTT (R = 2), respectively. Moreover, Table 1 shows that choosing R = 2 instead of R = 1 increases the sample size of SSD by approximately 12% and the ASN of the TT and DTT by the same proportion under H_0_, H_1 _and θ = θ_R_/2. On the other hand, as compared with SSD when R = 2 (SSD_adj_), there were decreases of approximately 46% and 29% under H_0_, 37% and 36% under H_1_, and 24% and 21% under θ = θ_R_/2 in the ASNs for the TT (R = 2) and DTT (R = 2), respectively.

**Table 1:** ASN required to reach a conclusion under H_0_/H_1_, and when θ = θ_R_/2 for the TT (R = 1), TT (R = 2), DTT (R = 1) and DTT (R = 2) and sample size for the SSD (R = 1) and SSD (R = 2), for different values of θ_R_, β (α = 0.05) and n = 12.

θ_R_	β	SSD R = 1	SSD R = 2	TT(R = 1)		TT(R = 2)		DTT(R = 1)		DTT(R = 2)	
				
				ASN (H_0_/H_1_)	ASN (θ_R_/2)	ASN (H_0_/H_1_)	ASN (θ_R_/2)	ASN (H_0_/H_1_)	ASN (θ_R_/2)	ASN (H_0_/H_1_)	ASN (θ_R_/2)
0.4	0.05	325	366	168/185	239	188/208	269	220/184	247	248/208	273
	0.1	263	296	135/164	191	152/184	215	179/165	199	201/185	224
0.5	0.05	208	234	108/120	155	122/134	173	143/120	159	160/135	179
	0.1	168	189	88/107	124	98/120	139	116/108	129	129/120	145
0.6	0.05	144	163	77/85	109	86/95	122	100/86	112	113/95	125
	0.1	117	131	62/76	87	69/85	98	82/76	90	92/85	102
0.7	0.05	106	119	57/64	81	64/71	91	75/64	84	84/72	93
	0.1	86	97	47/57	65	52/64	73	61/57	68	68/64	76
0.8	0.05	81	91	45/50	63	50/56	70	59/50	65	63/56	71
	0.1	66	74	37/45	51	41/50	57	46/44	51	53/50	59
0.9	0.05	64	72	36/41	51	40/45	56	46/41	51	52/45	58
	0.1	52	58	30/37	41	33/41	46	39/37	42	43/41	48
1.0	0.05	52	58	30/34	42	34/38	47	39/35	43	44/38	48
	0.1	42	47	25/31	34	28/34	38	29/29	32	36/34	39

**Figure 3:**
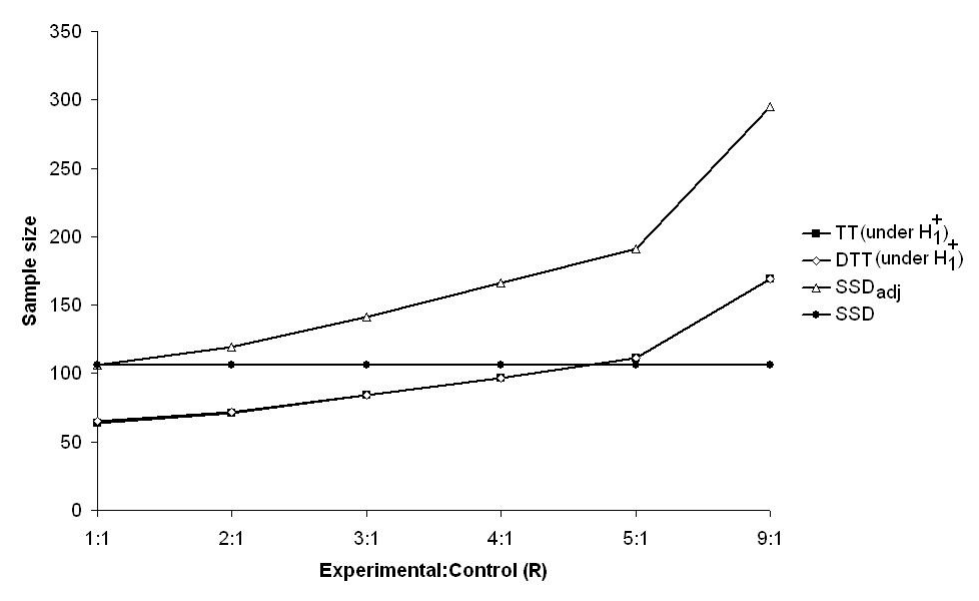
Sample size required by two-sided SSD and SSD_adj _and average sample number for the TT and the DTT (under H1+
 MathType@MTEF@5@5@+=feaafiart1ev1aaatCvAUfKttLearuWrP9MDH5MBPbIqV92AaeXatLxBI9gBaebbnrfifHhDYfgasaacH8akY=wiFfYdH8Gipec8Eeeu0xXdbba9frFj0=OqFfea0dXdd9vqai=hGuQ8kuc9pgc9s8qqaq=dirpe0xb9q8qiLsFr0=vr0=vr0dc8meaabaqaciaacaGaaeqabaqabeGadaaakeaacqqGibasdaqhaaWcbaGaeeymaedabaGaee4kaScaaaaa@2FB6@), for different values of R, when n = 12, θ_R _= 0.7 and β = α = 0.05.

Figure 3 displays the sample size required by the SSD and the ASN for SSD_adj_, TT (n = 12) and DTT (n = 12) as a function of R when θ_R _= 0.7 and β = 0.05. The ASN curves for the TT and DTT under H1+
 MathType@MTEF@5@5@+=feaafiart1ev1aaatCvAUfKttLearuWrP9MDH5MBPbIqV92AaeXatLxBI9gBaebbnrfifHhDYfgasaacH8akY=wiFfYdH8Gipec8Eeeu0xXdbba9frFj0=OqFfea0dXdd9vqai=hGuQ8kuc9pgc9s8qqaq=dirpe0xb9q8qiLsFr0=vr0=vr0dc8meaabaqaciaacaGaaeqabaqabeGadaaakeaacqqGibasdaqhaaWcbaGaeeymaedabaGaee4kaScaaaaa@2FB6@ always stayed beneath the sample size required by the SSD_adj _and were similar to one another. Indeed, as compared with SSD_adj_, there were decreases of approximately 39.5%, 39.5%, 40.4%, 41.5%, 42% and 42.7% in ASNs for TT and DTT when R was equal to 1, 2, 3, 4, 5 and 9, respectively. Also, for R ≤ 4, the ASN curves of the TT and DTT stayed beneath the sample size required by the SSD (N = 106). Indeed, as compared with SSD, for R ≤ 4, they were decreased by approximately 39.5%, 32%, 21% and 8.5% in ASNs for TT and DTT when R was equal to 1, 2, 3 and 4 respectively. On the other hand, for R ≥ 2 the ASN curve of SSD_adj _remained above the sample size required by SSD. Indeed, as compared with SSD, they were increased by approximately 12.3%, 33%, 56.6%, 80% and 178% in ASNs for SSD_adj _when R was equal to 2,3,4,5 and 9, respectively.

**Figure 4:**
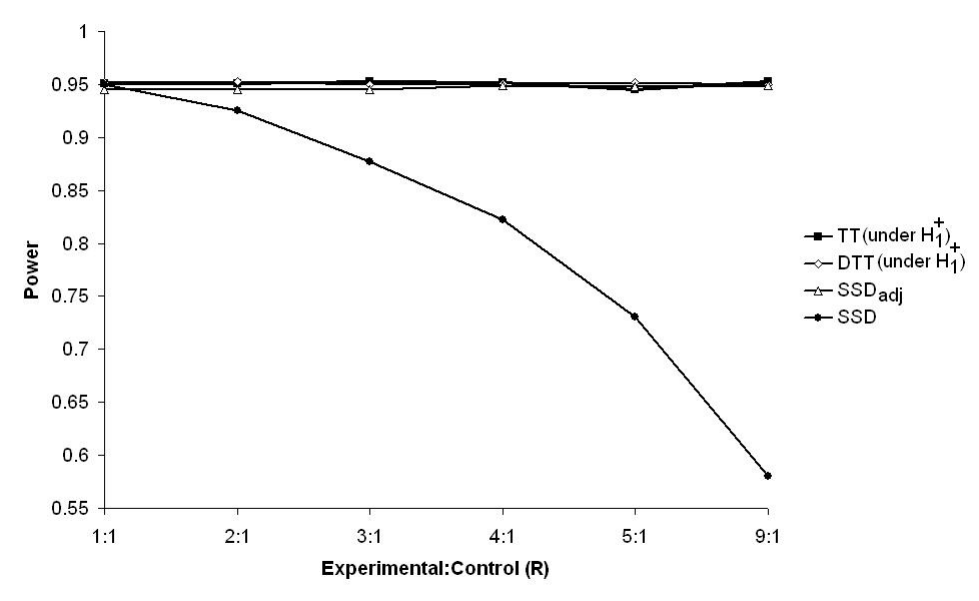
Exact power for the SSD, and estimated power for the TT, the DTT (under H1+
 MathType@MTEF@5@5@+=feaafiart1ev1aaatCvAUfKttLearuWrP9MDH5MBPbIqV92AaeXatLxBI9gBaebbnrfifHhDYfgasaacH8akY=wiFfYdH8Gipec8Eeeu0xXdbba9frFj0=OqFfea0dXdd9vqai=hGuQ8kuc9pgc9s8qqaq=dirpe0xb9q8qiLsFr0=vr0=vr0dc8meaabaqaciaacaGaaeqabaqabeGadaaakeaacqqGibasdaqhaaWcbaGaeeymaedabaGaee4kaScaaaaa@2FB6@) and the SSD_adj _for different values of R, when n = 12, θ_R _= 0.7 and β = α = 0.05.

Figure 4 shows that in the SSD, with a fixed total sample size, unequal randomization can lead to a reduction in statistical power when the R increases. In contrast, for the TT, DTT and SSD_adj _where there is no limit on patient recruitment, the power curves remain constant.

## Discussion

Sequential methods and unequal randomization design are two different techniques in clinical trials, with their ethical and economic advantages. However, no previous study has combined unequal randomization with sequential analyses. In other words, the debates concerning unequal randomization were restricted to the SSD [11-16] and sequential analyses were only discussed in situations where the patients were equally randomized between the treatment groups [7,8]. Sebille and Bellissant [8] showed that, of the one-sided sequential tests, the one-sided TT (R = 1) offers a substantial decrease in sample size compared with the one-sided SSD (R = 1); namely 40% under H_0 _and H_1 _and 25% under θ = θ_R_/2. In addition, they showed that the two-sided TT (R = 1) offers a two-sided conclusion with much fewer patients than the double TT (R = 1) and two-sided SSD (R = 1), but at the expense of a high decrease in power under H1-
 MathType@MTEF@5@5@+=feaafiart1ev1aaatCvAUfKttLearuWrP9MDH5MBPbIqV92AaeXatLxBI9gBaebbnrfifHhDYfgasaacH8akY=wiFfYdH8Gipec8Eeeu0xXdbba9frFj0=OqFfea0dXdd9vqai=hGuQ8kuc9pgc9s8qqaq=dirpe0xb9q8qiLsFr0=vr0=vr0dc8meaabaqaciaacaGaaeqabaqabeGadaaakeaacqqGibasdaqhaaWcbaGaeeymaedabaGaeeyla0caaaaa@2FBA@[7]. On the other hand, according to Avins [13], Edwards [14] and Pocock [16], unbalanced randomization has ethical advantages since more patients are randomized to what is thought to be the superior therapy. Also, Torgerson and Campbell [11,12] showed that when research costs differ between treatments and there is no constraint on total sample size, it is more cost-effective to randomize more patients to the less expensive treatment. However, Pocock [16] showed that, when there is a ceiling on total sample size, unequal randomization leads to a reduction in statistical power. Hence, we expected that, in the practical situation, in which the sample size of the SSD cannot be adjusted, the TT (R = 2) and DTT (R = 2), compared with unadjusted SSD, decrease the sample size while maintaining the power of the trial to its planned value. As an important result, our simulation study showed that even with the maximum ASN which occurs at θ = θ_R_/2, the TT (R = 2) and DTT (R = 2) have smaller ASNs than the SSD (R = 1). Also before the start of the work, we could not estimate how much using R = 2 instead of R = 1 would increase the sample size in the TT, DTT and SSD. However, based on our findings, choosing R = 2 instead of R = 1 equally increases the sample size in the sequential methods and SSD up to 12%. Nevertheless, when the costs of the two treatment groups are very different, allocation of more patients to the cheaper treatment in the TT and DTT will compensate for this increase rate in the sample size. This decreases the total cost of the trial substantially.

However, it is necessary to present some characteristics of our study. Firstly, to present a fair comparison, we have only evaluated the statistical properties of the two-sided TT and DTT under H_0_, H1+
 MathType@MTEF@5@5@+=feaafiart1ev1aaatCvAUfKttLearuWrP9MDH5MBPbIqV92AaeXatLxBI9gBaebbnrfifHhDYfgasaacH8akY=wiFfYdH8Gipec8Eeeu0xXdbba9frFj0=OqFfea0dXdd9vqai=hGuQ8kuc9pgc9s8qqaq=dirpe0xb9q8qiLsFr0=vr0=vr0dc8meaabaqaciaacaGaaeqabaqabeGadaaakeaacqqGibasdaqhaaWcbaGaeeymaedabaGaee4kaScaaaaa@2FB6@ and θ_R_/2, because, under these hypotheses, their power functions are identical [7]. Secondly, we did not compare the one-sided TT with other two-sided tests simultaneously because it is quite controversial in the literature [18,19].

## Conclusion

This study shows that if we allocate patients unequally in the SSD among the treatment groups and sample size ceiling cannot be increased to maintain the power of the trial due to economic restrictions, then an amalgamation of the sequential analysis and unequal randomization, compared with SSD, can be a compromise between statistical, ethical and economic requirements.

## Competing interests

The author(s) declare that they have no competing interests.

## Authors' contributions

PJ was responsible for the design, simulations, analyses and interpretation. SMTA and JB contributed to the analyses and interpretation.

## Pre-publication history

The pre-publication history for this paper can be accessed here:


